# HuR‐regulated lncRNA NEAT1 stability in tumorigenesis and progression of ovarian cancer

**DOI:** 10.1002/cam4.710

**Published:** 2016-04-14

**Authors:** Yiqing Chai, Jie Liu, Zhikun Zhang, Liwei Liu

**Affiliations:** ^1^ The Ultrasound CentreTianjin central hospital of gynecology obstetricsTianjin300052China; ^2^ The Department of UrologyThe Second hospital of Tianjin medical universityTianjin300211China

**Keywords:** HuR, lncRNA, miR‐124, NEAT1, ovarian cancer

## Abstract

Long noncoding RNAs (lncRNAs) have recently emerged as pivotal regulators in governing fundamental biological processes, as well as in tumorigenesis. The nuclear paraspeckle assembly transcript 1 (NEAT1) is one of the most highly regulated lncRNAs in recent genomic datasets, however, its biological role and regulatory mechanism in ovarian cancer (OC) development and progression are poorly defined. In this study, we identified that NEAT1 was up‐regulated in OC patients and cell lines, and its expression was associated with the FIGO stage and lymph node metastasis. Furthermore, the ectopic expression of NEAT1_1 in OVCAR‐3 cell lines promoted cell proliferation and invasion, whereas knockdown of NEAT1_1 did the opposite. Furthermore, NEAT1_1 was stabilized by an RNA‐binding protein HuR, but suppressed by miR‐124‐3p in OC cells. Accordingly, the increased HuR mRNA and decreased miR‐124‐3p levels were observed in OC patients. These results suggested that lncRNA NEAT1, whose expression was collaboratively controlled by HuR and miR‐124‐3p, could regulate ovarian carcinogenesis and may serve as a potential target for antineoplastic therapies.

## Introduction

Ovarian cancer (OC) is the most lethal gynecological cancer and a common cause of cancer‐related deaths in women worldwide 1, 2. Despite advances in surgery and chemotherapy, the overall survival of OC remains low. Therefore, a deep understanding of the molecular mechanisms implicated in ovarian carcinogenesis is required for the development of OC prevention, diagnosis, and therapy.

Recent studies have revealed that a number of lncRNAs have essential roles in a diverse range of cellular processes such as proliferation, differentiation, apoptosis, and cell fate as well as disease pathogenesis, causing a paradigm change in our understanding of gene regulation networks 3, 4, 5. For example, lncRNA HOX antisense intergenic RNA (HOTAIR) has been found to be dysregulated in a variety of cancers, such as urothelial carcinoma, pancreatic tumors, hepatocellular carcinoma, colorectal carcinomas, and OC 6, 7, 8, 9, 10. Metastasis‐associated lung adenocarcinoma transcript 1 (MALAT1), also named nuclear‐enriched abundant transcript 2 (NEAT2), is a widely expressed lncRNA that was first identified as a factor indicating high metastatic potential and poor prognosis in non‐small‐cell lung cancer (NSCLC) 11, 12, 13, 14. Recently, MALAT1 has been associated with several human neoplasms including lung, liver, renal, colorectal, gastric, and breast cancers 15, 16. Maternally expressed gene 3 (MEG3) also encodes an lncRNA, which is lost or significantly reduced in neuroblastomas, hepatocellular cancers, gastric cancer, and gliomas 17, 18. Therefore, identification of differential expression is the first step in the elucidation of lncRNA‐based molecular mechanisms capable of regulating tumorigenesis.

The nuclear paraspeckle assembly transcript 1 (NEAT1) is one of the most highly regulated lncRNAs in recent genomic datasets 19, 20. NEAT1 is transcribed from the familial tumor syndrome multiple endocrine neoplasia type 1 locus, which has two isoforms, 3.7 kb NEAT1_1 and 23 kb NEAT1_2 19. The alternation of NEAT1 level has been reported in different types of human malignancies, including leukemia, colorectal cancer, glioma, and hepatocellular carcinoma 20, 21, 22, 23. Given the essential nature of NEAT1 function in different human carcinomas, we decided to investigate its role in human OC. In this study, we perform RT‐qPCR analysis to detect the NEAT1 level in 65 paired OC tissues and adjacent normal tissues. Elevated NEAT1 levels were observed in OC patients, compared with the levels detected in adjacent normal tissues. We further identified that the up‐regulation of NEAT1 in OC was mediated by HuR, which binds to NEAT1 and increases the steady‐state levels of NEAT1. In contrast, a small noncoding RNA, miR‐124‐3p, directly targets NEAT1 and reduces its expression level in OC cells. Taken together, the expression of NEAT1 was controlled by two regulators, HuR and miR‐124‐2p, respectively. In ovarian carcinogenesis, increased HuR and decreased miR‐124‐3p collaboratively contributed to the overexpression of NEAT1. We also demonstrated that inhibition of NEAT1 (NEAT1_1) could reduce OC cell growth and progression. Collectively, our results may identify NEAT1 as a novel modulator of ovarian carcinogenesis, and illustrate a regulatory networks including HuR, miR‐124‐3p, and NEAT1.

## Materials and Methods

### Ovarian cancer tissues

Ovarian cancer tissues and their paired normal tissues (located >1 cm away from the tumor) were obtained between May 2012 and April 2015 from 65 OC patients undergoing surgery at the Tianjin central hospital of gynecology obstetrics. Tissue samples were immediately snap‐frozen in liquid nitrogen, and stored in liquid nitrogen until RNA extraction. The use of the tissue samples for all experiments was approved by all the patients and by Ethics Committee of the institution.

### Cell cultures and cell transfection

The OC cells lines used in this study were purchased from the Cell Resource Center of IBMS, CAMS. All cells were grown in RPMI 1640 media supplemented with 10% FBS and penicillin/streptomycin, and cultured in a 5% CO2 humidified incubator. The OVCAR‐3 cell lines were transfected with plasmid constructs as a final concentration of 2 *μ*g/mL using Lipo2000 (Invitrogen, CA, USA) in accordance with the manufacturer's instructions, and transfected with siRNA, siRNA control, miR‐124‐3p mimic, negative scramble control (GenePharma, Shanghai, China) at a final concentration of 25 nmol/L using DharmaFECT 1 (Dharmacon; Lafayette, CO) in accordance with the manufacturer's instructions.

### Tissue RNA isolation and RT‐qPCR

Total RNA was extracted from the cells and tissues using Trizol reagent (Invitrogen, CA, USA), according to the manufacturer's instructions. RT‐qPCR assay was conducted to detect the level of RNA transcripts. Briefly, cDNA was synthesized by M‐MLV reverse transcriptase (Invitrogen) from 3 *μ*g of total RNA. Oligo (dT18) RT primer was used for the reverse transcription of lncRNA and mRNA. Stem‐poop RT primer was used for the reverse transcription of miR‐124‐3p. RT‐qPCR was performed on the Bio‐rad CFX96 real‐time PCR System (Bio‐rad, CA, USA) using KAPA PROBE FAST qPCR Kits (Kapa Biosystems, MA, USA) and TaqMan probes (Invitrogen). The miR‐124‐3p‐specific forward primer sequence was designed on the basis of miRNA sequences obtained from the miRBase database. Human GAPDH and U6 snRNA were used for mRNA/lncRNA and miRNA normalization, respectively.

### Luciferase reporter assay

The sequence from NEAT1 that containing the putative miR‐124‐3p‐binding sites was PCR amplified and cloned into pGL3 to generate the corresponding reporters. A mutation in the miR‐124‐3p‐binding site sequence was created using the QuickChange SiteDirected Mutagenesis kit (Stratagene, CA, USA). For luciferase reporter analysis, the 293T cells were co‐transfected with 0.4 *μ*g of the reporter construct, 0.02 *μ*g of pRL‐TK control vector, and 5 pmol of miR‐124‐3p mimic or scramble controls. Cells were harvested 48 h post‐transfection and assayed with Dual Luciferase Assay (Promega, WI, USA) according to manufacturer's instructions. All transfection assays were carried out in triplicates.

### Cell proliferation assay

OVCAR‐3 cells were incubated in 10% CCK‐8 (DOJINDO, Japan) diluted in normal culture medium at 37°C until visual color conversion occurred. Proliferation rates were determined at 0, 12, 24, 48, 72, and 96 h after transfection. The absorbance of each well was measured with a microplate reader set at 450 nmol/L and 630 nmol/L. All experiments were performed in triplicate.

### Cell cycle analysis

OVCAR‐3 cells were collected and washed one time by putting 1 × 10^6^ cells per tube, adding 1 mL of PBS, and centrifuging at 600 g at 4°C. Resuspend pelleted cells in 0.3 mL of PBS buffer and add 0.7 mL cold ethanol (70%) dropwise to tube to fix the cells. Leave the cells on ice for 1 h (or up to a few days at 4°C), and centrifuge the cells as above, wash one time with cold PBS and recentrifuge. Add 10 *μ*L of 1 mg/mL PI solution (the final concentration being 10 *μ*g/mL) and 5 *μ*L of 10 mg/mL Rnase A (the final concentration being 0.2 mg/mL). Keep the cells in dark and at a temperature of 4°C until analysis. Analyze on FACS by reading on cytometer at 488 nm.

### Cell invasion assays

OVCAR‐3 cells were grown to confluence on 12‐well plastic dishes and treated with plasmid constructs pCMV6‐NEAT1_1 and empty pCMV6. After 24 h transfection, 1 × 10^5^ OVCAR‐3 cells in serum‐free media were seeded onto the transwell migration chambers (8 *μ*m pore size; Millipore, Switzerland) which coated with the upper chamber of an insert coated with Matrigel (Sigma‐Aldrich, St Louis, MO). Media containing 20% FBS were added to the lower chamber. After 24 h, the noninvading cells were removed with cotton wool, invasive cells located on the lower surface of the chamber were stained with May‐Grunwald‐Giemsa stain (Sigma‐Aldrich, USA) and counted using a microscope (Olympus, Tokyo, Japan). All experiments were performed in triplicate.

### RNA Immunoprecipitation (RIP) assays

OVCAR‐3 cells were collected and washed one time by PBS, and the pellets were resuspended in freshly prepared RIP buffer, kept on ice for 20 min and centrifuging at 10,000 g for 10 min. Anti‐HuR antibodies were added to (5 *μ*g) to the supernatant and incubate for 4 h at 4°C with gentle rotation. Add protein A beads (40 *μ*L) and incubate for another 2 h at 4°C with gentle rotation. Pellet beads at 1600 g for 30 sec, remove the supernatant, and resuspend beads in 500 mL RIP buffer. Repeat for a total of three RIP washes, followed by one wash in PBS. Isolate coprecipitated RNAs by resuspending beads in TRIzol RNA extraction reagent (1 mL) according to manufacturer's instructions.

### RNA pull‐down by MS2‐MBP

Maltose‐binding protein (MBP)‐affinity purification was used to identify RNA‐binding proteins (RBPs) that were associated with NEAT1. The MS2‐MBP protein was expressed and purified from *E. coli* following a protocol from the Steitz lab. Three bacteriophage MS2 coat protein‐binding sites (5′‐cgtacaccatcagggtacgagctagcccatggcgtacaccatcagggtacgactagtagatctcgtacaccatcagggtacg‐3′) were inserted downstream of NEAT1_1 by site‐directed mutagenesis using Stratagene's QuikChange Site‐Directed Mutagenesis Kit. To obtain proteins associated with Ms2‐tagged NEAT1_1, OVCAR‐3 cells were transfected with pCMV6‐NEAT1_1‐MS2 constructs for 48 h, and 1 × 10^7^ cells were used for each immunoprecipitation assay.

### Western blot analysis

Immunoblotting analysis was carried out using standard methods. Proteins were separated by 10% SDS‐PAGE, and transferred onto PVDF membranes (Millipore Corporation, Billerica MA, USA). Membranes were blocked overnight with 5% nonfat dried milk for 2 h and incubated with anti‐HuR antibody (Abcam, ab54987) at 1:1000 dilution; anti‐GAPDH antibody (Proteintech) at 1:50,000 dilution overnight at 4°C. After washing with TBST (10 mmol/L Tris, pH 8.0, 150 mmol/L NaCl, and 0.1% Tween20), the membranes were incubated for 2 h at room temperature with goat anti‐mouse antibody (Zsgb‐bio, Beijing, China) at 1:20000.

### Statistical analyses

Student's *t*‐test (two‐tailed) was performed to analyze the data. A two‐sided *P*‐value of less than 0.05 was considered statistically significant. All statistical computations were performed using SPSS (SPSS Inc., Chicago, IL, USA).

## Results

### Aberrant expression of lncRNA NEAT1 in human OC tissues and cell lines

**Figure 1:**
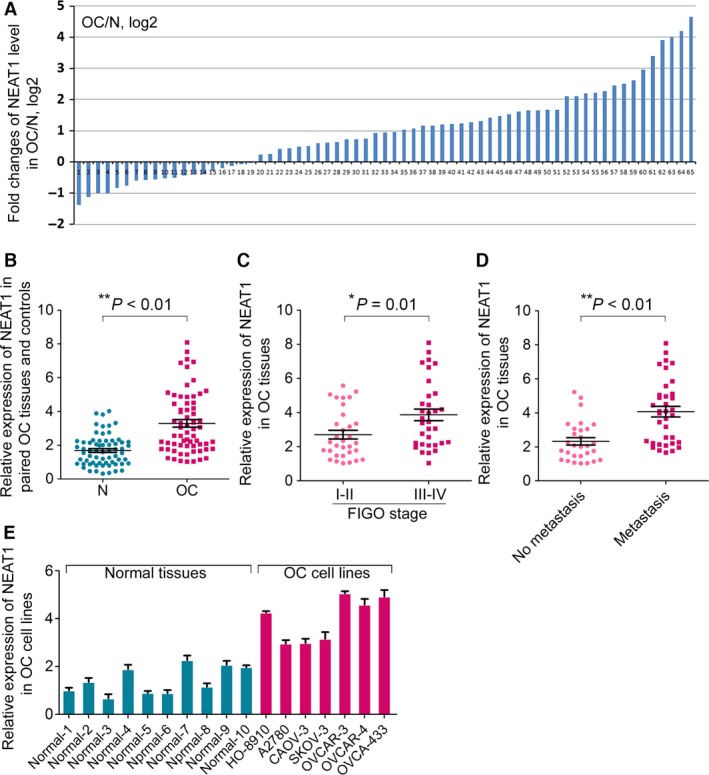
The expression of lncRNA NEAT1 in ovarian cancer (OC) tissues and OC cell lines. (A) The expression level of NEAT1 was detected in 65 pairs of OC patients by RT‐qPCR. Data were presented as fold change in OC tissues relative to adjacent normal regions. (B) Relative NEAT1 expression level in OC tissues and adjacent normal regions. (C) The Statistical analysis of the association between NEAT1 level and FIGO stage. (D) The Statistical analysis of the association between NEAT1 level and lymph node metastasis. (E) The relative level of NEAT1 in seven OC cell lines relative to ten normal control samples. For all quantitative results, the data are presented as the mean ± SEM, and the error bars represent the standard deviation obtained from three independent experiments. *, *P *<* *0.05; **, *P *<* *0.01.

To determine the expression level of lncRNA NEAT1 in OC, RT‐qPCR analysis using TaqMan probes was conducted in 65 pairs of clinic OC tissue and matched adjacent normal tissue samples, and normalized to ACTB. Among them, 49 cases (~75%) showed significantly increased level (>1.5‐fold) of NEAT1 in tumor tissues compared with their normal control, whereas only 10 cases (~15%) showed down‐regulated NEAT1 level (>1.5‐fold) in OC samples (Fig. 1A). In summary, the level of NEAT1 was significantly increased in OC tissue compared with adjacent normal tissues (Fig. 1B). Next, the clinicopathological significance of NEAT1 expression in OC was analyzed. Interestingly, we found that the higher NEAT1 was positively correlated with the FIGO stage of tumor (Fig. 1C) and the occurrence of lymph node metastasis (Fig. 1D), but not with other clinicopathological variables (Table S1). These results indicated that the dysregulation of NEAT1 in OC patients might suggest a potential oncogenic role of NEAT1 in ovarian carcinogenesis.

### NEAT1 promotes OC cell proliferation and invasion

**Figure 2:**
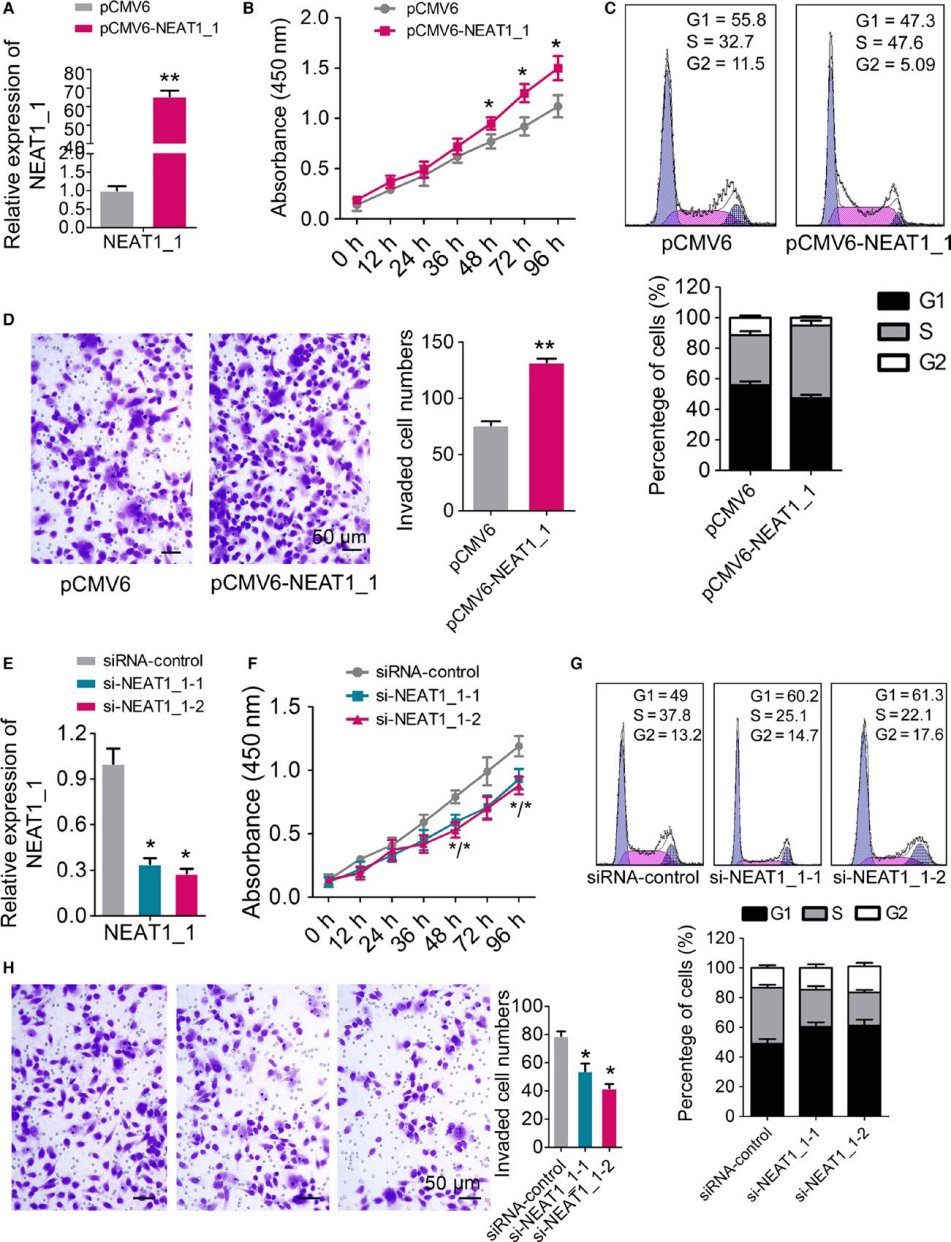
The gain‐ and loss‐of‐function analysis of NEAT1_1 in ovarian cancer cells. (A) NEAT1_1 level was detected in OVCAR‐3 cells after treatment with pCMV6‐NEAT1_1 or pCMV6 empty vector by RT‐qPCR. (B) Cell proliferation assay of OVCAR‐3 cells after treatment with pCMV6‐NEAT1_1 or pCMV6 by using CCK‐8. (C) Flow cytometry was applied to examine the cell cycle progression of OVCAR‐3 cells after treatment with pCMV6‐NEAT1_1 or pCMV6 and stained with PI. The percentage of cells was shown in the lower panel. (D) Transwell analysis of OVCAR‐3 cells after treatment with pCMV6‐NEAT1_1 or pCMV6, the relative ratio of invasive cells per field was shown right. (E) NEAT1_1 level were detected in OVCAR‐3 cells after treatment with si‐NEAT1_1 or siRNA controls by RT‐qPCR. (F) Cell proliferation assay of OVCAR‐3 cells after treatment with si‐NEAT1_1 or siRNA controls by using CCK‐8. (G) Flow cytometry was applied to examine the cell cycle progression of OVCAR‐3 cells after treatment with si‐NEAT1_1 or siRNA controls and stained with PI. The percentage of cells was shown in the lower panel. (H) Transwell analysis of OVCAR‐3 cells after treatment with si‐NEAT1_1 or siRNA controls, the relative ratio of invasive cells per field was shown right. For all quantitative results, the data are presented as the mean ± SEM, and the error bars represent the standard deviation obtained from three independent experiments. *, *P *<* *0.05; **, *P *<* *0.01.

To investigate the potential role of NEAT1 in OC progression, we first detected the relative levels of NEAT1 in seven different OC cell lines, including HO‐8901, A2780, CAOV‐3, SKOV‐3, OVCAR‐3, OVCAR‐4, and OVCA‐433. Because NEAT1 was found to be expressed at the highest level in OVCAR‐3 (Fig. 1E), we chose this cell line for further analysis. A construct containing the 3.7 kb NEAT1 isoform (pCMV‐NEAT1_1) was transfected into the OVCAR‐3 cells and the efficiency of NEAT1_1 overexpression was subsequently confirmed by RT‐qPCR (Fig. 2A). The ectopic expression of NEAT1_1 led to significantly increased proliferation compared with the empty vector‐transfected OVCAR‐3 cells (Fig. 2B). Accordingly, flow cytometric analysis revealed that the percentage of S phase cells was also increased by ~15% in OVCAR‐3 cells upon NEAT1_1 overexpression (Fig. 2C). Next, transwell assays showed increased number of OVCAR‐3 cells in lower section in the NEAT1_1 overexpressing groups (Fig. 2D).

In contrast, two sets of siRNAs specific to NEAT1 (si‐NEAT1_1‐1 and si‐NEAT1_1‐2) were transfected into OVCAR‐3 cells to further test whether this lncRNA was functionally involved in OC tumorigenesis. The intracellular level of NEAT1 was reduced by threefold and fourfold in OVCAR‐3 cells treated with NEAT1_1 siRNAs compared to the siRNA controls (Fig. 2E). Cell proliferation of OVCAR‐3 cells was inhibited in NEAT1_1 knockdown groups (Fig. 2F), and the cell cycle progression was also retarded (Fig. 2G). In addition, reduced expression of NEAT1_1 dramatically inhibited the normally strong invasive capacity of OVCAR‐3 cells as indicated in the transwell invasion assay (Fig. 2H). Taken together, these results suggest that NEAT1_1 might act as an oncogene through promoting cell proliferation and invasion in OC cells.

### NEAT1 is physically associated with and stabilized by HuR

**Figure 3:**
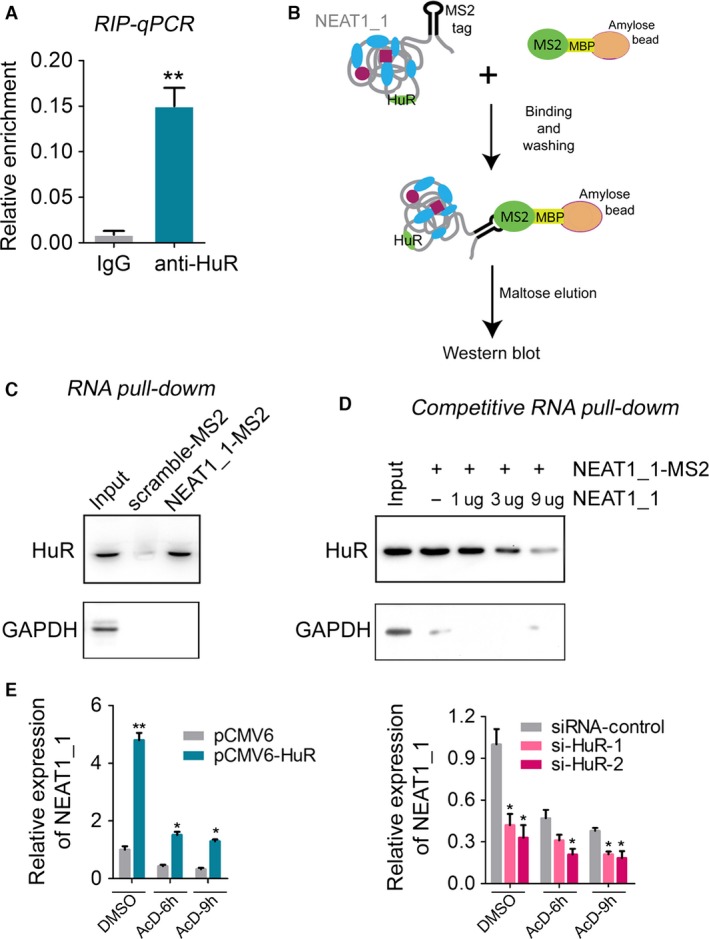
NEAT1_1 was associated with and regulated by HuR. (A) The RT‐qPCR analysis of coprecipitated RNAs in RNA immunoprecipitation (RIP) assay. IgG was used as a negative control in RIP assay. (B) A Schematic model of RNA pull‐down assay. (C) RNA pull‐down assay followed by western blot to detect HuR endogenously associated with MS2‐tagged NEAT1_1. (D) Competitive RNA pull‐down assay followed by western blot to detect HuR endogenously associated with MS2‐tagged NEAT1_1 after co‐transfection with non tagged‐NEAT1_1. (E) NEAT1_1 level was detected by RT‐qPCR in OVCAR‐3 cells transfected with pCMV6‐HuR or pCMV6 (the left panel), and si‐HuR or siRNA controls (the right panel) for 48 h, these cells were followed by AcD treatment for indicated times. For all quantitative results, the data are presented as the mean ± SEM, and the error bars represent the standard deviation obtained from three independent experiments. *, *P *<* *0.05; **, *P *<* *0.01.

Based on the above findings that NEAT1 participated in ovarian carcinogenesis, we decided to explore the regulatory mechanism controlling NEAT1 expression in OC cells. A previous study has indicated the interaction between HuR and NEAT1 RNA in a genome‐wide screen for site‐specific interactions between RNA‐binding factors and total RNA called PAR‐CLIP 24. Therefore, we first tested the interaction between HuR and NEAT1 in OC cells by RNA‐immunoprecipitation (RNA‐IP). RIP lysate prepared from OVCAR‐3 cells were immunoprecipitated by anti‐HuR antibody or mouse IgG, and the co‐precipitated RNA was validated by RT‐qPCR. As shown in Figure 3A, the endogenous NEAT1 transcripts were specially associated with HuR in OVCAR‐3 cells. Additionally, to further validate the association between HuR and NEAT1_1, we performed an RNA pull‐down analysis to isolate the endogenous HuR associated with NEAT1_1 (Fig. 3B). The precipitated proteins were analyzed by immunoblotting and results showed that the MS2‐tagged NEAT1_1 (NEAT1_1‐MS2) in OVCAR‐3 cells was significantly enriched for HuR compared to the MS2‐tagged scrambles (scramble‐MS2) (Fig. 3C). The irrelevant protein GAPDH was used as a negative control. Furthermore, the interaction specificity was further examined by a competitive RNA pull‐down experiment. The binding of HuR to MS2‐tagged NEAT1_1 was efficiently competed by increasing amounts of non‐MS2‐tagged NEAT1_1 (Fig. 3D). Taken together, these data suggested that NEAT1_1 RNA represents a target for HuR in OC cells.

Since the human RBP HuR is a conserved mRNA stability regulator 19, we next detected whether HuR could influence the stability of NEAT1_1 RNA in actinomycin D (AcD)‐treated OVCAR‐3 cells. As expected, overexpression of HuR resulted in twofold to threefold increase in NEAT1_1 level after cell transcription was blocked by AcD (Fig. 3E). In contrast, a significant decrease in NEAT1_1 level was observed in OVCAR‐3 cells with HuR knockdown (Fig. 3E). In summary, these data demonstrated that HuR could bind to NEAT1 and increase its stability.

### Expression of HuR is up‐regulated in OC tissues

**Figure 4:**
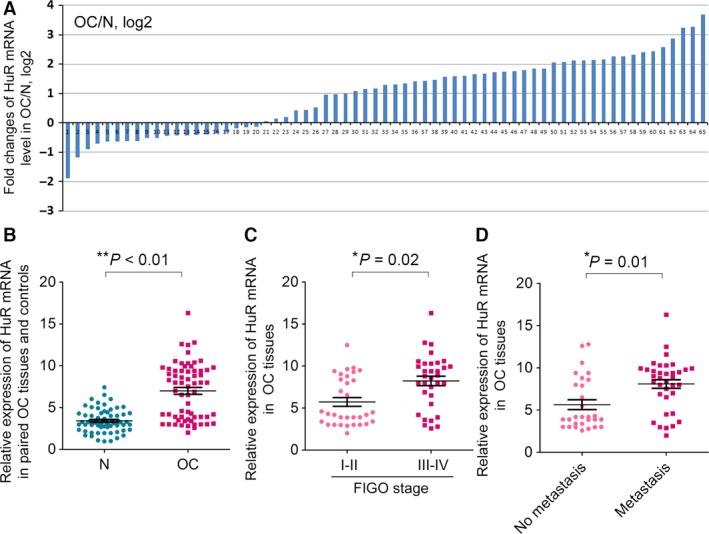
The expression of HuR in ovarian cancer (OC) tissues. (A) The expression level of HuR mRNA was detected in 65 pairs of OC patients by RT‐qPCR. Data were presented as fold change in OC tissues relative to adjacent normal regions. (B) Relative HuR mRNA expression level in OC tissues and adjacent normal regions. (C) The Statistical analysis of the association between HuR mRNA level and FIGO stage. (D) The Statistical analysis of the association between HuR mRNA level and lymph node metastasis. For all quantitative results, the data are presented as the mean ± SEM, and the error bars represent the standard deviation obtained from three independent experiments. *, *P *<* *0.05; **, *P *<* *0.01.

Given the regulatory roles of HuR on NEAT1 expression, we continued to detect whether the expression of HuR was also altered in OC tissues. RT‐qPCR was first used to measure the level of HuR mRNA in above 65 pairs of OC samples and showed the up‐regulation of HuR mRNA in ~73% of them (Fig. 4A). In summary, HuR mRNA was significantly increased in OC tissues compared with adjacent normal tissues (Fig. 4B). Moreover, the higher HuR mRNA level was also positively correlated with the FIGO stage of tumor (Fig. 4C) and the occurrence of lymph node metastasis (Fig. 4D). Therefore, HuR may also play an important role in ovarian carcinogenesis.

### NEAT1 is a target of miR‐124‐3p

**Figure 5:**
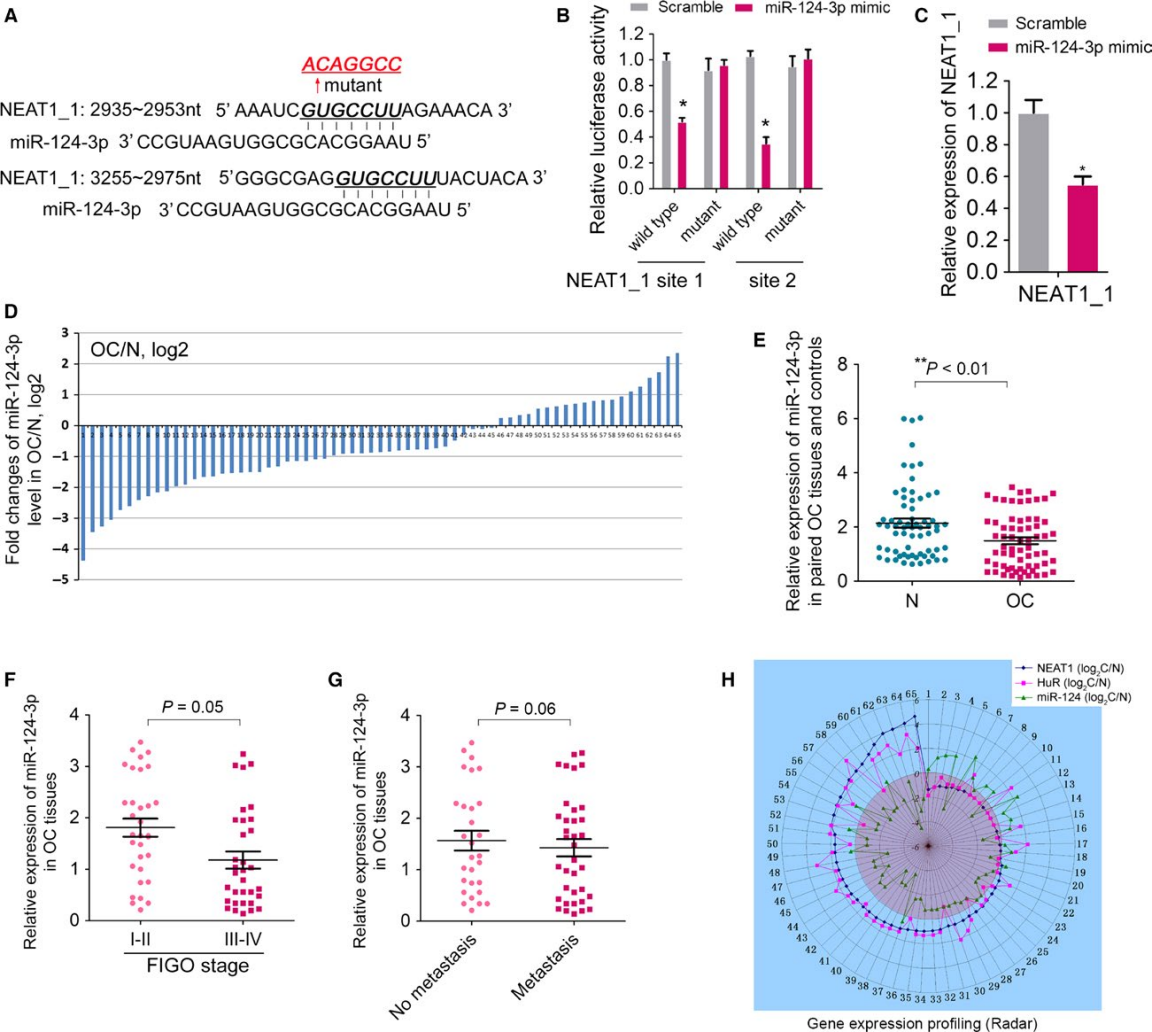
NEAT1_1 was repressed by miR‐124‐3p in ovarian cancer (OC) cells. (A) A computer prediction of the conserved and mutated binding sites within human NEAT1_1 transcripts for miR‐124‐3p. (B) Relative luciferase activity of the indicated NEAT1_1 reporter constructs. Error bars represent the standard deviation obtained from three independent experiments. (C) RT‐qPCR analysis of NEAT1_1 in OVCAR‐3 cells transfected with miR‐124‐3p mimic or scramble controls. (D) The expression of level of miR‐124‐3p was detected in 65 pairs of OC patients by RT‐qPCR. Data were presented as fold change in OC tissues relative to adjacent normal regions. (E) Relative miR‐124‐3p expression level in OC tissues and adjacent normal regions. (F) The Statistical analysis of the association between miR‐124‐3p level and FIGO stage. (G) The Statistical analysis of the association between miR‐124‐3p level and lymph node metastasis. (H) The normalized relative expression of NEAT1, HuR mRNA and miR‐124‐3p from the 65 pairs of OC samples were plotted in a radar graph on a logarithmic scale. For all quantitative results, the data are presented as the mean ± SEM, and the error bars represent the standard deviation obtained from three independent experiments. *, *P *<* *0.05; **, *P *<* *0.01.

Although the RNA stability was largely affected by such RBPs, the recent competing endogenous RNA (ceRNA) hypothesis posits that specific lncRNA could be targeted by specific sets of miRNAs. Thus, we began to search for potential miRNAs which target NEAT1. After alignment, we found two putative binding site of miR‐124‐3p in the NEAT1_1 transcripts (Fig. 5A). To validate whether miR‐124‐3p regulates NEAT1_1, we first cloned the wild‐type or miR‐124‐3p‐binding site‐mutant NEAT1_1 (Fig. 5A) into a pMIR‐reporter plasmid and co‐transfected these constructs into 293T cells with an miR‐124‐3p mimic or a negative control (scramble), respectively. Reporter assays in 293T cells revealed that miR‐124‐3p significantly reduced the luciferase activities of wild‐type NEAT1_1 reporters compared to the scramble (Fig. 5B). In contrast, the luciferase activities of the mutant reporters were not repressed by miR‐124‐3p, indicating that the repression was dependent on miRNA binding (Fig. 5B). Moreover, RT‐qPCR assay was carried out in OVCAR‐3 cells and showed that NEAT1_1 was about twofold lower in cells transfected with miR‐124‐3p mimics (Fig. 5C).

Accordingly, the down‐regulation of miR‐124‐3p was seen in patients with OC compared to the normal tissue controls (*P *<* *0.01) (Fig. 5D and E). However, there was no statistical significance of miR‐124‐3p level with the FIGO stage (Fig. 5F) and the occurrence of lymph node metastasis (Fig. 5G).

Taken together, a positive relationship between HuR and NEAT1 expression was observed in above OC samples, whereas the miR‐124‐3p level was negatively correlated with NEAT1 expression in OC (Fig. 5H).

## Discussion

Paraspeckles are unique subnuclear structures that are built around a specific long noncoding RNA (lncRNA), NEAT1, which is comprised of two isoforms (NEAT1_1 and NEAT1_2) that are produced by alternative 3′‐end processing 25. The NEAT1_1 and NEAT1_2 isoforms ratio dynamically changes in a cell type‐specific manner. In adult mouse tissues, NEAT1_2 is expressed in limited cell types, such as the epithelial layers of digestive tissues. By contrast, NEAT1_1 is expressed in a broader range of cell types 25. After its finding, the biological role of NEAT1 was mainly related to hypoxia, viral infection as well as human embryonic stem cells differentiation 21, 26, 27. Recently, the levels of NEAT1 are reported to be dynamically regulated in different cancer types. For example, the plasma NEAT1 level could act as fingerprint in NSCLC 23. NEAT1 expression in colorectal cancer was up‐regulated and related to tumor differentiation, invasion, metastasis, and TNM stage 22. Moreover, the high NEAT1 expression was shown to be an independent prognostic marker of poor outcome in colorectal cancer 22. Besides these findings, NEAT1 was also involved in hepatocellular carcinoma, chronic lymphocytic leukemia, and prostate cancer 20, 28, 29. However, its involvement in ovarian tumorigenesis has not been well defined. In that regard, it would be interesting to test the biological and mechanistic significance of NEAT1 in OC. In general, our observations corroborated that NEAT1 was an oncogenic regulator for human OC cells. Therefore, its regulation in cancer along with the development of new therapeutic targets of NEAT1 may represent promising tools against OC therapy.

Post‐transcriptional regulation through specific RBPs is emerging as a critical regulating level in nearly all the biological processes. As a kind of RBPs, HuR plays important role in stabilizing and/or modulating the translation of many of its target mRNAs including p21, c‐fos, VEGF, MKP‐1, TNF‐*α*, Bcl‐2, Mcl‐1, and p53 30. The exact mechanisms by which HuR stabilized these mRNAs are not fully defined, but HuR likely competed with other RBPs that might lead to their recruitment to the exosome for mRNA degradation 30. Through its influence on specific target mRNAs, HuR can alter multiple cellular responses to proliferative, stress, apoptotic, differentiation, senescence, inflammatory, and immune stimuli 31. Recently, HuR is increasingly recognized as a pivotal factor in cancer‐related gene expression. This function is based on HuR's ability to promote the expression of proteins that enhance proliferation, inhibit apoptosis, increase angiogenesis, and facilitate invasion and metastasis 30. Meanwhile, HuR has also been the focus of several studies in OC 32, 33. Consistent with these studies, our results also indicated the oncogenic role of HuR in OC. However, unlike these studies, we focused on its control of an lncRNA rather than protein‐coding mRNAs in tumorigenesis. Specifically, we identified lncRNA NEAT1 as a novel target of HuR in OC cells. In agreement with this influence, we observed enhanced NEAT1 expression upon HuR overexpression, whereas reduced NEAT1 level when HuR was inhibited. In addition, we also found the significantly increased HuR mRNA expression in OC patients, which was consistent with NEAT1's oncogenic role in OC. Thus, HuR might be involved in the progression of ovarian carcinogenesis via modulating NEAT1 expression.

Most diseases, including human cancer, are frequently associated with an altered transcription pattern 34. The past few years have witnessed an exciting increase in the richness and complexity of RNA‐mediated regulatory circuitries including new types of RNA–RNA interaction, especially the interaction between miRNAs and lncRNAs via complementary base pairing 35. MiR‐124‐3p is the most abundant miRNA in the brain, and the biogenesis of miR‐124‐3p displays specific temporal and spatial profiles in various cell and tissue types 36. Recently, the link between dysregulation of miR‐124‐3p and different tumors has become evident 37, 38. However, the relationship between miR‐124‐3p and OC had not been reported. In this study, we found that miR‐124‐3p is markedly decreased in OC patients, and its tumor suppressive roles might due to its regulation on NEAT1 expression.

Taken together, our research demonstrated that NEAT1 acts as a key regulator in human ovarian carcinogenesis and revealed two regulatory factors (HuR and miR‐124‐3p) of NEAT1 expression. In ovarian carcinogenesis, there is a close connection between the up‐regulation of HuR, down‐regulation of miR‐124‐3p, and NEAT1. These results have significant implications regarding our understanding of OC pathogenesis. Moreover, the pleiotropic effects of NEAT1 on OC tumorigenesis suggest that it could be an effective target for antineoplastic therapies.

## Conflict of Interest

The authors declare that neither financial nor non‐financial competing interests exist.

## Supporting information


**Table S1.** Association of NEAT1, HuR and miR‐124‐3p expression with the clinicopathological variables in OC.

## References

[1] Raspaglio, G. , I. De Maria , F. Filippetti , E. Martinelli , G. F. Zannoni , S. Prislei , et al. 2010 HuR regulates beta‐tubulin isotype expression in ovarian cancer. Cancer Res. 70:5891–5900. 20587520 10.1158/0008-5472.CAN-09-4656

[2] Cheng, Z. , J. Guo , L. Chen , N. Luo , W. Yang , and X. Qu . 2015 A long noncoding RNA AB073614 promotes tumorigenesis and predicts poor prognosis in ovarian cancer. Oncotarget 6:25381–25389. 26299803 10.18632/oncotarget.4541 PMC4694838

[3] Wilusz, J. E. 2016 Long noncoding RNAs: re‐writing dogmas of RNA processing and stability. Biochim. Biophys. Acta 1859:128–138. 26073320 10.1016/j.bbagrm.2015.06.003 PMC4676738

[4] Zhu, J. , H. Fu , Y. Wu , and X. Zheng . 2013 Function of lncRNAs and approaches to lncRNA‐protein interactions. Sci. China Life Sci. 56:876–885. 24091684 10.1007/s11427-013-4553-6

[5] Bassett, A. R. , A. Akhtar , D. P. Barlow , A. P. Bird , N. Brockdorff , D. Duboule , et al. 2014 Considerations when investigating lncRNA function in vivo. eLife 3:e03058. 25124674 10.7554/eLife.03058 PMC4132285

[6] Qiu, J. J. , Y. Wang , J. X. Ding , H. Y. Jin , G. Yang , and K. Q. Hua . 2015 The long non‐coding RNA HOTAIR promotes the proliferation of serous ovarian cancer cells through the regulation of cell cycle arrest and apoptosis. Exp. Cell Res. 333:238–248. 25796453 10.1016/j.yexcr.2015.03.005

[7] Bhan, A. , and S. S. Mandal . 2015 LncRNA HOTAIR: a master regulator of chromatin dynamics and cancer. Biochim. Biophys. Acta 1856:151–164. 26208723 10.1016/j.bbcan.2015.07.001 PMC4544839

[8] Li, H. , J. An , M. Wu , Q. Zheng , X. Gui , T. Li , et al. 2015 LncRNA HOTAIR promotes human liver cancer stem cell malignant growth through downregulation of SETD2. Oncotarget 6:27847–27864. 26172293 10.18632/oncotarget.4443 PMC4695030

[9] Loewen, G. , J. Jayawickramarajah , Y. Zhuo , and B. Shan . 2014 Functions of lncRNA HOTAIR in lung cancer. J. Hematol. Oncol. 7:90. 25491133 10.1186/s13045-014-0090-4 PMC4266198

[10] Zhang, A. , J. C. Zhao , J. Kim , K. W. Fong , Y. A. Yang , D. Chakravarti , et al. 2015 LncRNA HOTAIR enhances the androgen‐receptor‐mediated transcriptional program and drives castration‐resistant prostate cancer. Cell Rep. 13:209–221. 26411689 10.1016/j.celrep.2015.08.069 PMC4757469

[11] Dong, Y. , G. Liang , B. Yuan , C. Yang , R. Gao , and X. Zhou . 2015 MALAT1 promotes the proliferation and metastasis of osteosarcoma cells by activating the PI3K/Akt pathway. Tumour Biol. 36:1477–1486. 25431257 10.1007/s13277-014-2631-4

[12] Weber, D. G. , G. Johnen , S. Casjens , O. Bryk , B. Pesch , K. H. Jockel , et al. 2013 Evaluation of long noncoding RNA MALAT1 as a candidate blood‐based biomarker for the diagnosis of non‐small cell lung cancer. BMC Res. Notes 6:518. 24313945 10.1186/1756-0500-6-518 PMC4029199

[13] Guo, F. , F. Jiao , Z. Song , S. Li , B. Liu , H. Yang , et al. 2015 Regulation of MALAT1 expression by TDP43 controls the migration and invasion of non‐small cell lung cancer cells in vitro. Biochem. Biophys. Res. Commun. 465:293–298. 26265046 10.1016/j.bbrc.2015.08.027

[14] Wei, Y. , and B. Niu . 2015 Role of MALAT1 as a prognostic factor for survival in various cancers: a systematic review of the literature with meta‐analysis. Dis. Markers 2015:164635. 26420912 10.1155/2015/164635 PMC4572489

[15] Sheng, X. , J. Li , L. Yang , Z. Chen , Q. Zhao , L. Tan , et al. 2014 Promoter hypermethylation influences the suppressive role of maternally expressed 3, a long non‐coding RNA, in the development of epithelial ovarian cancer. Oncol. Rep. 32:277–285. 24859196 10.3892/or.2014.3208

[16] Yin, D. D. , Z. J. Liu , E. Zhang , R. Kong , Z. H. Zhang , and R. H. Guo . 2015 Decreased expression of long noncoding RNA MEG3 affects cell proliferation and predicts a poor prognosis in patients with colorectal cancer. Tumour Biol. 36:4851–4859. 25636452 10.1007/s13277-015-3139-2

[17] Peng, W. , S. Si , Q. Zhang , C. Li , F. Zhao , F. Wang , et al. 2015 Long non‐coding RNA MEG3 functions as a competing endogenous RNA to regulate gastric cancer progression. J. Exp. Clin. Cancer Res. 34:79. 26253106 10.1186/s13046-015-0197-7 PMC4529701

[18] Sun, M. , R. Xia , F. Jin , T. Xu , Z. Liu , W. De , et al. 2014 Downregulated long noncoding RNA MEG3 is associated with poor prognosis and promotes cell proliferation in gastric cancer. Tumour Biol. 35:1065–1073. 24006224 10.1007/s13277-013-1142-z

[19] Zhen, L. , L. Yun‐Hui , D. Hong‐Yu , M. Jun , and Y. Yi‐Long . 2015 Long noncoding RNA NEAT1 promotes glioma pathogenesis by regulating miR‐449b‐5p/c‐Met axis. Tumour Biol. [Epub ahead of print]. 10.1007/s13277-015-3843-y 26242266

[20] Chakravarty, D. , A. Sboner , S. S. Nair , E. Giannopoulou , R. Li , S. Hennig , et al. 2014 The oestrogen receptor alpha‐regulated lncRNA NEAT1 is a critical modulator of prostate cancer. Nat. Commun. 5:5383. 25415230 10.1038/ncomms6383 PMC4241506

[21] Choudhry, H. , A. Albukhari , M. Morotti , S. Haider , D. Moralli , J. Smythies , et al. 2015 Tumor hypoxia induces nuclear paraspeckle formation through HIF‐2alpha dependent transcriptional activation of NEAT1 leading to cancer cell survival. Oncogene 34:4546. 26289678 10.1038/onc.2014.431 PMC7609283

[22] Li, Y. , Y. Li , W. Chen , F. He , Z. Tan , J. Zheng , et al. 2015 NEAT expression is associated with tumor recurrence and unfavorable prognosis in colorectal cancer. Oncotarget 6:27641–27650. 26314847 10.18632/oncotarget.4737 PMC4695014

[23] Hu, X. , J. Bao , Z. Wang , Z. Zhang , P. Gu , F. Tao , et al. 2015 The plasma lncRNA acting as fingerprint in non‐small‐cell lung cancer. Tumour Biol. [Epub ahead of print]. 10.1007/s13277-015-4023-9 26453113

[24] Lebedeva, S. , M. Jens , K. Theil , B. Schwanhausser , M. Selbach , M. Landthaler , et al. 2011 Transcriptome‐wide analysis of regulatory interactions of the RNA‐binding protein HuR. Mol. Cell 43:340–352. 21723171 10.1016/j.molcel.2011.06.008

[25] Naganuma, T. , and T. Hirose . 2013 Paraspeckle formation during the biogenesis of long non‐coding RNAs. RNA Biol. 10:456–461. 23324609 10.4161/rna.23547 PMC3672290

[26] Imamura, K. , N. Imamachi , G. Akizuki , M. Kumakura , A. Kawaguchi , K. Nagata , et al. 2014 Long noncoding RNA NEAT1‐dependent SFPQ relocation from promoter region to paraspeckle mediates IL8 expression upon immune stimuli. Mol. Cell 53:393–406. 24507715 10.1016/j.molcel.2014.01.009

[27] Standaert, L. , C. Adriaens , E. Radaelli , A. Van Keymeulen , C. Blanpain , T. Hirose , et al. 2014 The long noncoding RNA Neat1 is required for mammary gland development and lactation. RNA 20:1844–1849. 25316907 10.1261/rna.047332.114 PMC4238351

[28] Blume, C. J. , A. Hotz‐Wagenblatt , J. Hullein , L. Sellner , A. Jethwa , T. Stolz , et al. 2015 p53‐dependent non‐coding RNA networks in chronic lymphocytic leukemia. Leukemia 29:2015–2023. 25971364 10.1038/leu.2015.119

[29] Guo, S. , W. Chen , Y. Luo , F. Ren , T. Zhong , M. Rong , et al. 2015 Clinical implication of long non‐coding RNA NEAT1 expression in hepatocellular carcinoma patients. Int. J. Clin. Exp. Pathol. 8:5395–5402. 26191242 PMC4503113

[30] Abdelmohsen, K. , and M. Gorospe . 2010 Posttranscriptional regulation of cancer traits by HuR. Wiley Interdiscip. Rev. RNA 1:214–229. 21935886 10.1002/wrna.4 PMC3808850

[31] Srikantan, S. , and M. Gorospe . 2012 HuR function in disease. Front Biosci. 17:189–205. 10.2741/3921 PMC4540328 22201738

[32] Denkert, C. , W. Weichert , S. Pest , I. Koch , D. Licht , M. Köbel , et al. 2004 Overexpression of the embryonic‐lethal abnormal vision‐like protein HuR in ovarian carcinoma is a prognostic factor and is associated with increased cyclooxygenase 2 expression. Cancer Res. 64:189–195. 14729623 10.1158/0008-5472.can-03-1987

[33] Davidson, B. , A. Holth , E. Hellesylt , R. Hadar , B. Katz , C. G. Tropé , et al. 2016 HUR mRNA expression in ovarian high‐grade serous carcinoma effusions is associated with poor survival. Hum. Pathol. 48:95–101. 26640230 10.1016/j.humpath.2015.09.027

[34] Liz, J. , and M. Esteller . 2016 lncRNAs and microRNAs with a role in cancer development. Biochim. Biophys. Acta 1859:169–176. 26149773 10.1016/j.bbagrm.2015.06.015

[35] Guil, S. , and M. Esteller . 2015 RNA‐RNA interactions in gene regulation: the coding and noncoding players. Trends Biochem. Sci. 40:248–256. 25818326 10.1016/j.tibs.2015.03.001

[36] Sun, Y. , Z. M. Luo , X. M. Guo , D. F. Su , and X. Liu . 2015 An updated role of microRNA‐124 in central nervous system disorders: a review. Front. Cell. Neurosci. 9:193. 26041995 10.3389/fncel.2015.00193 PMC4438253

[37] Arabkheradmand, A. , A. Safari , M. Seifoleslami , E. Yahaghi , and M. Gity . 2015 Down‐regulated microRNA‐124 expression as predictive biomarker and its prognostic significance with clinicopathological features in breast cancer patients. Diagn. Pathol. 10:178. 26415857 10.1186/s13000-015-0391-0 PMC4587828

[38] Taniguchi, K. , N. Sugito , M. Kumazaki , H. Shinohara , N. Yamada , Y. Nakagawa , et al. 2015 MicroRNA‐124 inhibits cancer cell growth through PTB1/PKM1/PKM2 feedback cascade in colorectal cancer. Cancer Lett. 363:17–27. 25818238 10.1016/j.canlet.2015.03.026

