# Supplementary material for: HuR‐regulated lncRNA NEAT1 stability in tumorigenesis and progression of ovarian cancer
Source: Cancer Med. 2016 Apr 14;5(7):1588–98. doi: 10.1002/cam4.710 (PMC4944886; doi:10.1002/cam4.710)
Supplement: Supplementary file 1 — Table S1. Association of NEAT1, HuR and miR‐124‐3p expression with the clinicopathological variables in OC. [file CAM4-5-1588-s001.doc]

**Supplemental Table S1. Association of NEAT1, HuR and miR-124-3p expression with the clinicopathological variables in OC.**

| **Variables** | **N** | **NEAT1**  **Mean** ± SD | ***P*** | **HuR mRNA**  **Mean** ± SD | ***P*** | **miR-124-3p**  **Mean** ± SD | ***P*** |
| --- | --- | --- | --- | --- | --- | --- | --- |
|  |  |  |  |  |  |  |  |
| **Age, years** |  |  | 0.27 |  | 0.12 |  | 0.24 |
| ≥50 | 42 | 3.18 ± 1.67 |  | 6.62 ± 3.12 |  | 1.54 ± 1.09 |  |
| <50 | 23 | 3.49 ± 2.04 |  | 7.68 ± 3.55 |  | 1.35 ± 1.02 |  |
| **Histological subtype** |  |  | 0.27 |  | 0.21 |  | 0.09 |
| Serous | 49 | 3.31 ± 1.77 |  | 6.80 ± 3.27 |  | 1.56 ± 1.07 |  |
| Other | 16 | 3.54 ± 1.93 |  | 7.60 ± 3.41 |  | 1.20 ± 0.85 |  |
| **FIGO stage** |  |  | 0.01* |  | 0.02* |  | 0.05 |
| FIGO I-II | 32 | 2.70 ± 1.43 |  | 5.73 ± 2.89 |  | 1.81 ± 1.00 |  |
| FIGO III-IV | 33 | 3.86 ± 2.0 |  | 8.37 ± 3.17 |  | 1.20 ± 0.95 |  |
| **Lymph node metastasis** |  |  | <0.01* |  | 0.01* |  | 0.06 |
| Absent | 29 | 2.32 ± 1.16 |  | 5.64 ± 3.10 |  | 1.56 ± 1.03 |  |
| Present | 37 | 4.05 ± 1.90 |  | 8.30 ± 3.02 |  | 1.43 ± 0.99 |  |
| **Histological grade** |  |  | 0.08 |  | 0.06 |  | 0.11 |
| G1–G2 | 29 | 2.31 ± 1.41 |  | 5.69 ± 2.19 |  | 1.55 ± 0.98 |  |
| G3 | 36 | 4.06 ± 2.02 |  | 8.40 ± 3.35 |  | 1.45 ± 1.01 |  |
| **CA125 level (U/ml)** |  |  | 0.49 |  | 0.28 |  | 0.48 |
| <600 | 25 | 3.29 ± 1.86 |  | 6.82 ± 3.43 |  | 1.48 ± 1.041 |  |
| ≥600 | 40 | 3.30 ± 1.74 |  | 7.29 ± 3.11 |  | 1.46 ± 1.01 |  |

1. Mean±SD, mean of C/N, Standard Definition of C/N. C, normalized expression of cancer tissues; N, normalized expression of adjacent noncancerous tissues.
2. * Indicated statistical significance (*P*<0.05).
